# Maximum Growth Potential and Periods of Resource Limitation in Apple Tree

**DOI:** 10.3389/fpls.2016.00233

**Published:** 2016-02-29

**Authors:** Francesco Reyes, Theodore DeJong, Pietro Franceschi, Massimo Tagliavini, Damiano Gianelle

**Affiliations:** ^1^Forests and Biogeochemical Cycles, Department of Sustainable Agro-Ecosystems and Bioresources, Centro Ricerca e Innovazione – Fondazione Edmund MachSan Michele all’Adige, Italy; ^2^Faculty of Science and Technology, Free University of Bolzano-BozenBolzano, Italy; ^3^Department of Plant Science, University of California at Davis, DavisCA, USA; ^4^Department of Computational Biology, Centro Ricerca e Innovazione – Fondazione Edmund MachSan Michele all’Adige, Italy

**Keywords:** maximum potential growth, relative growth rate, carbon allocation, source/sink, vegetative growth, fruit growth, tree growth, shoot growth

## Abstract

Knowledge of seasonal maximum potential growth rates are important for assessing periods of resource limitations in fruit tree species. In this study we assessed the periods of resource limitation for vegetative (current year stems, and woody biomass) and reproductive (fruit) organs of a major agricultural crop: the apple tree. This was done by comparing relative growth rates (RGRs) of individual organs in trees with reduced competition for resources to trees grown under standard field conditions. Special attention was dedicated to disentangling patterns and values of maximum potential growth for each organ type. The period of resource limitation for vegetative growth was much longer than in another fruit tree species (peach): from late May until harvest. Two periods of resource limitation were highlighted for fruit: from the beginning of the season until mid-June, and about 1 month prior to harvest. By investigating the variability in individual organs growth we identified substantial differences in RGRs among different shoot categories (proleptic and epicormic) and within each group of monitored organs. Qualitatively different and more accurate values of growth rates for vegetative organs, compared to the use of the simple compartmental means, were estimated. Detailed, source-sink based tree growth models, commonly in need of fine parameter tuning, are expected to benefit from the results produced by these analyses.

## Introduction

The availability of carbohydrates is fundamental to plant growth, structural development, and crop yields. The growth of an individual plant component follows seasonal patterns, specific for each organ type, but also depends on competition for resources (such as carbohydrates, nitrogen, and water) with the other plant parts (Hansen, 1971; Grossman and DeJong, 1995b,c; Cheng and Fuchigami, 2002).

Early in the growing season, carbohydrate reserves stored in roots are thought to support respiration, but not growth, of the above ground biomass (Loescher et al., 1990; Lakso et al., 1999).

In the early growth stages, young organs can be viewed as ‘parasitic’ (Watson and Casper, 1984; Lauri et al., 2010), meaning that they exhibit heterotrophic growth based on imported assimilates. Hansen (1971) suggested that, in apple tree, more than one-half to two-thirds of assimilates for flowers (until they reach about 200 mg/spur) and for stems (until the first 5–6 leaves are formed, about 500–1000 mg/shoot) do not originate from current photosynthesis, but from carbon reserves present in the tree. Then, depending on type, the organ may become autonomous (autotrophic growth), producing most of the carbohydrates it needs. Eventually they can start to export assimilates: after attaining one-third to one-half of their final area in the case of leaves (Wardlaw, 1968) but in different proportions depending on the growth stage and position along the stem in the case of extension shoots (Hansen, 1967b). On the other hand, the apple fruits exhibit a largely heterotrophic growth, essentially based on the import of assimilates from proximate leaves (Hansen, 1967a, 1969, 1977). As such, fruit growth competes for local assimilates, as clearly suggested by the negative relationship between shoot secondary growth and the presence of an adjacent fruit (Lauri et al., 2010). High fruit loads are reported to strongly suppress vegetative growth in apple and citrus, and especially in alternate bearing varieties (Dudney, 1974; Hansen, 1977; Monselise and Goldschmidt, 1982; Martínez-Alcántara et al., 2015).

Higher starch concentrations in stems were found in lightly cropped tree structures compared to heavily cropped ones (Naschitz et al., 2010), and in years off (with low yield) compared to years on (with high yield) in apple trees (Monselise and Goldschmidt, 1982). A similar result was also found for total nitrogen and carbon in alternate bearing citrus trees (Martínez-Alcántara et al., 2015). Additionally, starch in stems was found to increase after harvest in apple (Naschitz et al., 2010). The higher availability of starch (Naschitz et al., 2010) and the increased secondary growth in shoots (Lauri et al., 2010) associated with low fruit loads or absence of adjacent fruit, suggest that fruits compete with shoots, by consuming the carbon available from nearby shoots.

Depending on the availability of carbon assimilates, and on the fruit load, growth of different organs can be limited by resource availability (source limited) or by genetically determined endogenous characteristics of the organ (sink limited). The RGR associated with sink limited organs is called maximum potential RGR (Wareing and Patrick, 1975; Grossman and DeJong, 1995b,c; Marcelis, 1996). Sink limited growth conditions of an organ can be approximated by maximizing the resources available to it, via manipulations such as selective removal of competing organs (heavy thinning, defruiting) in plants growing in optimal field conditions (Grossman and DeJong, 1995b,c, DeJong, 1998). Maximum potential RGR, and RGRs of organs in standard field conditions can then be compared in order to identify periods of source limited growth (DeJong, 1998), as was previously done for peach trees (Grossman and DeJong, 1995b,c).

In growth analysis, organ size is commonly described as a function of temperature. Temperature is indeed considered as the primary environmental factor affecting fruit growth, given non-limiting nutrient, and water conditions (Johnson and Lakso, 1985). For this reason a commonly used index for physiological time is the accumulation of GDDs, calculated as the summation of temperatures above a base temperature, starting from a specific phenological phase, e.g., full bloom. A base temperature of 5°C has been suggested for apple fruit growth (Warrington et al., 1999), while 4°C was used for apple shoot growth (Johnson and Lakso, 1985).

Apple orchards are a major agricultural crop worldwide, with over 60 million tons of apples produced on more than 7 million hectares per year (O’Rourke, 2003) and are the focus of a large amount of research to improve their production efficiency and sustainability (Lauri et al., 2009). In this context, an improved knowledge of the periods of resource limitation in apple could help to more accurately tune the timing of management practices, such as fruit thinning.

Resource limitations for vegetative and fruit growth in apple occur most likely in two periods. The first one is during the exponential phase of fruit growth, when fruits are weak sinks, and may be affected by competition after shoot tips (Quinlan and Preston, 1971). This period can last up to 2–4 weeks from bloom (Quinlan and Preston, 1968; Lakso and Corelli Grappadelli, 1998; Lakso et al., 1999), but ends before mid-June, when fruits become strong sinks (Hansen, 1977). The second period may occur during the 2 weeks before harvest and is thought to be related to declining incident light and cooler temperatures in the late season (Lakso and Corelli Grappadelli, 1998; Lakso et al., 1999).

The maximum potential RGR is also used to compute sink strength, the basic information needed to predict carbon partitioning in source-sink carbon allocation models. Sink strength is obtained by multiplying the maximum potential RGR by the sink size (Marcelis, 1996) and summing the result (potential net sink strength) with the potential maintenance and growth respiration rates. Because of its relevance in the process of carbon allocation, accurate knowledge of maximum potential RGR of different organs might allow to investigate, via in-silico experiments: (1) the tuning of practices such as fruit thinning, pruning and artificial spur extinction, (2) an optimal equilibrium between vegetative and fruit growth, and (3) an optimal yield and individual fruit size.

The current work focuses primarily on assessing the periods of resource limitation of different organ types in apple trees, such as fruits, stems of the vegetative shoots and trunk. This was done by comparing primary growth and dry weight accumulation of individual organs in heavily THI and DEF plants with plants growing under standard field conditions. Second, it is considered that even within a manipulated canopy, local conditions, such as shading, could lead to resource limitation for organ growth and, eventually, possible underestimates of the maximum RGR. As such, this study is also dedicated to the search for more accurate estimates of the maximum RGRs, taking into account the variability in individual organ growth.

## Materials and Methods

### Study Area

The study area was located in the floodplain of the Adige River, municipality of Caldaro, South Tyrol, Italy (46°21′ N, 11°16′ E; 240 m a.s.l.), a region extensively dedicated to intensive apple plantations. An organic apple orchard (*Malus domestica*, Fuji Variety grafted on M9 rootstock) planted in year 2000 was chosen as the study site. Distances among trees followed a regular scheme of 3 m between rows and 1 m along the row. Trees were trained as spindelbushes, mechanically THI and managed according to organic farming guidelines; pruning included tree topping at about 3.6 m. Drip irrigation was used throughout the growing season in order to prevent water stress. Analysis of total nitrogen, available phosphorus and exchangeable potassium showed optimal nutrients availability (**Supplementary Table S1**). An automated meteorological station on the same site recorded air temperature every 30 min. The mean annual temperature, in the period 1980–2010, was of 11.6°C (Zanotelli et al., 2013), slightly higher than in 2014 (12.8°C). Maximum leaf area index was reached in July. Average fruit yield per tree was higher in year 2014 (26.2 kg/tree) than in any of the previous years in the period 2009–2012 (**Table 1**).

**Table 1:** Apple fresh weight at harvest.

	Year	Fresh weight (Kg)	
FRU	2009 to 2012	Min	13.6
		Max	22.3
FRU	2014	Mean	26.2
		*SD*	6.2
THI	2014	Mean	6.2
		*SD*	7.1


*Yields per tree are reported for trees in standard field conditions (FRU) and heavily thinned trees (THI), in the year of the experiment (2014). The range of the average yearly yield for FRU trees at the orchard level is also reported for the period 2009–2012 (adapted from Zanotelli et al., 2013).*

### Experimental Design

**FIGURE 1:**
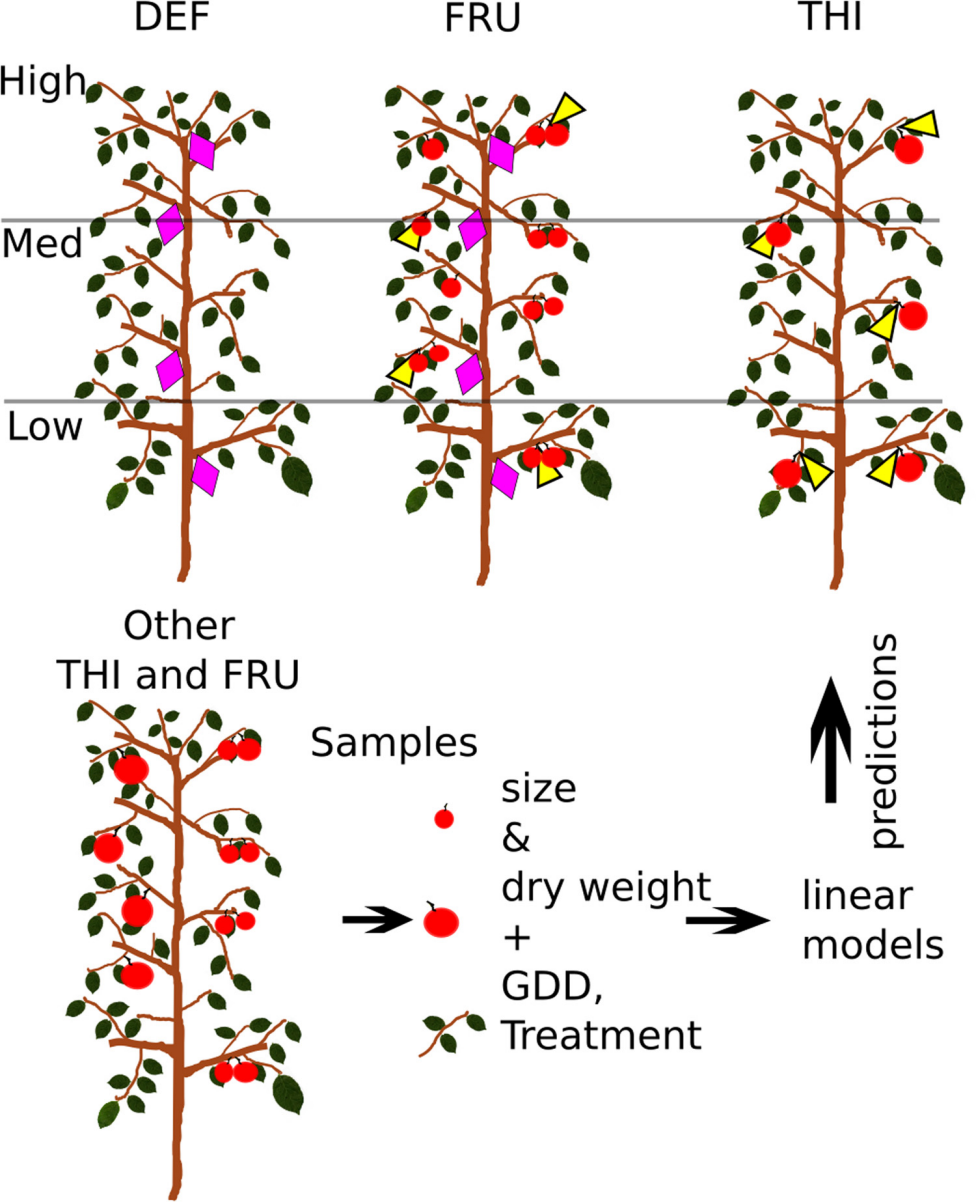
**Experimental design.** Shoots (violet diamonds) were tagged and measured on fruited (FRU) and defruited (DEF) trees, while fruits (yellow triangles) were measured on fruited and heavily thinned (THI) trees. Fruits and shoots were also sampled from other THI and FRU trees. Sizes, dry weights and treatment of sampled organs, and the growing degree days (GDD) of the date of sampling were used to build linear models, later used to estimate the dry weights of tagged organs.

Fifteen trees were selected and divided in three groups. Each group was manipulated in order to minimize the competition for carbohydrates among target organs and other plant components. Accordingly, all fruits were removed 3 weeks after bloom (on 23rd April, 150 GDD) in the first group of five trees (Defruited trees: DEF), so that vegetative growth (trunk, leaves, vegetative shoots) would not be limited by competition from fruits. Similarly, fruits from a second group of five trees (Thinned trees: THI) were heavily THI, leaving no more than one fruit/bourse shoot or spur. A third group of five trees (FRU) was not manipulated, carried an average of 0.47 fruits/shoot or spur and served as a control treatment. An additional five trees were manipulated as the THI treatment and later used for supplemental fruit sampling (**Figure 1**).

#### Shoot Growth

All the vegetative shoots of about 20% of the branches from each of three canopy levels (Low: 0 to 1.20 m above ground, Medium: 1.20 to 2.40 m, High: 2.40 to top of the canopy) on each FRU and DEF tree were tagged according to a stratified random sampling scheme (**Figure 1**). Shoot length was measured approximately every 2 weeks from April to July, and monthly in August and September 2014. In order to discriminate between branches potentially containing proleptic and epicormic shoots (see Materials and Methods – Shoot Elongation Shoots), traces of pruning were recorded for each branch on DEF trees (DeJong et al., 2012). Basal diameters of 10 shoots/tree were measured 1 cm above the insertion point on four dates during the season (mid-July, mid-August, early-September and in the following winter).

Additionally, a random sample of 30 vegetative shoots, stratified according to tree level, orientation (North/South) and shoot length, was also taken from ancillary, non-manipulated FRU trees on each of eight dates during years 2014 and 2015 (mid-April, early July, late August, early September 2014, and end of May, mid-June, early and late July 2015). Their lengths were measured, and shoot and fruit dry weights determined after oven drying at 70°. Eventually, 20 shoots were randomly sampled from each treatment at the end of the growing season, and used to calculate shoot density as the ratio of dry weight and volume, estimated by immersion of the shoots in water.

#### Trunk Growth

Trunk collars (1 mm precision) were installed at about 10 cm above the grafting point on each DEF and FRU tree, and trunk circumference were measured every two weeks starting approximately 7 weeks after full bloom.

#### Fruit Growth

Three orthogonal diameters of nine tagged fruits/tree were measured approximately weekly with a digital caliper on each of five FRU and THI trees. In addition, 15 fruits/date were uniformly sampled from the three canopy levels of other FRU and THI trees on alternate dates; their diameters were measured and dry weight determined after over drying at 70°C. Volume of all fruits was estimated by means of the formula for rotational spheroids (Volume = 4/3^∗^π^∗^r_1_^∗^r_2_^∗^r_3_). Dry weights of the monitored fruits were determined at the time of harvest, in early October.

### Data Analysis

All data were recorded on a Microsoft Excel spreadsheet and processed in the R environment (R Core Team, 2015). All data were analyzed using, as a time reference, the accumulated GDDs after full bloom, considered as a proxy for physiological time. This was calculated as the daily mean of the hourly mean temperatures minus 4.5°C, after cutoff of values outside the range 4.5–35°C.

#### Shoots

Only the tagged shoots that reached lengths longer than 4 cm by the end of the growing season were used for analysis, while the smaller ones were removed from the dataset as probable spurs.

A generalized multiple linear regression model (Faraway, 2005) was built to estimate the dry weight of the sampled shoots (DWshoot) (eq. 1, **Supplementary Figures S1**–**S3**) (RMSE = 0.546 g, Adj *R*^2^ = 0.959). This was preferred to a simple linear model because the dry weight of the sampled shoots did not follow a normal distribution. Based on the Akaike Informaion Criterion (AIC) (Akaike, 1992), the shoot lengths, logarithm of shoot lengths and GDD were chosen as predictor variables.

DWshoot=shoot⁢ length+log⁡(shoot⁢ length)*GDD

The model was built for FRU, however, considering some allometric relationship (see Shoot Growth) it was applied to predict the dry weight of all shoots, independent of the treatment, knowing that the biomasses obtained for DEF shoots were lower estimates than their actual values.

#### Trunk

Dry weight of the woody biomass of the tree structures, excluding current year vegetative shoots, was estimated by means of an allometric relationship (eq. 2) (*R*^2^ = 0.91), previously established for trees of the same orchard (Zanotelli et al., 2013), relating the trunk circumference to total above-ground woody biomass (wood_AGB_), i.e., as after winter pruning.

$${wood}_{AGB}=202.9379*{\left(\frac{cfr}{\pi }\right)}^{1.6115}$$

#### Fruit

A generalized linear model was built to estimate the dry weights of the sampled fruit (DWapple) (eq. 3, **Supplementary Figures S4**–**S6**) (RMSE = 2.364 g, Adj *R*^2^ = 0.994), and then applied to estimate the dry weights of the tagged fruits. Based on AIC, the logarithm of the estimated volumes, GDD and treatment were chosen as predictor variables:

DWapple=log⁡(Volume)+Treatment*GDD

#### Relative Growth Rates and Maximum Potential Growth

The increases in shoot length, and shoot, fruit and trunk dry mass, and the GDD values between successive measurement dates were used to calculate RERs and RGR of these organs, following the classical approach to growth analysis (Causton and Venus, 1981). Then, differences in shoot RER and fruit RGR between treatments were detected for each measurement date by applying, respectively, one-sided unpaired Mann–Whitney tests and *t*-tests, adjusted for multiple comparison with Bonferroni correction.

Despite the removal of organs potentially competing for resources, a relatively large variability was observed in the growth of individual organs in the DEF and THI trees in this experiment. In order to identify the maximum potential growth rates for each organ type, while accounting for the observed variability, we proceeded as follows. Shoots were first split in two categories: as epicormic shoots most commonly grow as a consequence of severe pruning, shoots present on branches with traces of pruning were considered as potentially epicormic, all others were treated as proleptics. From each category, a small group of the five shoots that reached the highest dry masses by the end of the growing season were then used to represent the maximum potential RER and RGR of proleptic and epicormic shoots.

The same approach was not as appropriate for identifying the curve of maximum potential growth for fruit. Indeed, a large part of the observed variability in fruit weight at harvest for THI was found significantly (*p* < 0.001) related to the fruit weight at the beginning of the season (**Supplementary Figure S7**). The dry weight on the first measurement date, in turn, was negatively related (also significantly, *p* < 0.001) to the early season RGR of the fruits (**Supplementary Figure S8**). This can be interpreted as follows: as fruit RGR sharply decreases in the early growth stages, the low RGR associated to relatively high masses at the beginning of the season (for THI fruits) suggests that much of the observed variability in RGR and dry masses for THI was simply due to differential times of fruit set. As such, in the case of fruit the simple mean THI was preferred to a sub-population of THI to represent the maximum potential growth.

Regarding the woody biomass, a multivariate linear regression model was built to assess if treatment and GDD were significant factors affecting the relative increment in trunk circumference. Since this was the case, straight lines were fitted through the means of the logarithmically transformed above ground biomass data vs. GDD for each treatment, and for the tree with the highest increment in normalized circumference through the season (Max). The slopes of these straight lines represent the RGR of the wood_AGB_ of the trees (Hunt, 1982; Grossman and DeJong, 1995c).

## Results

### Shoot Elongation

**FIGURE 2:**
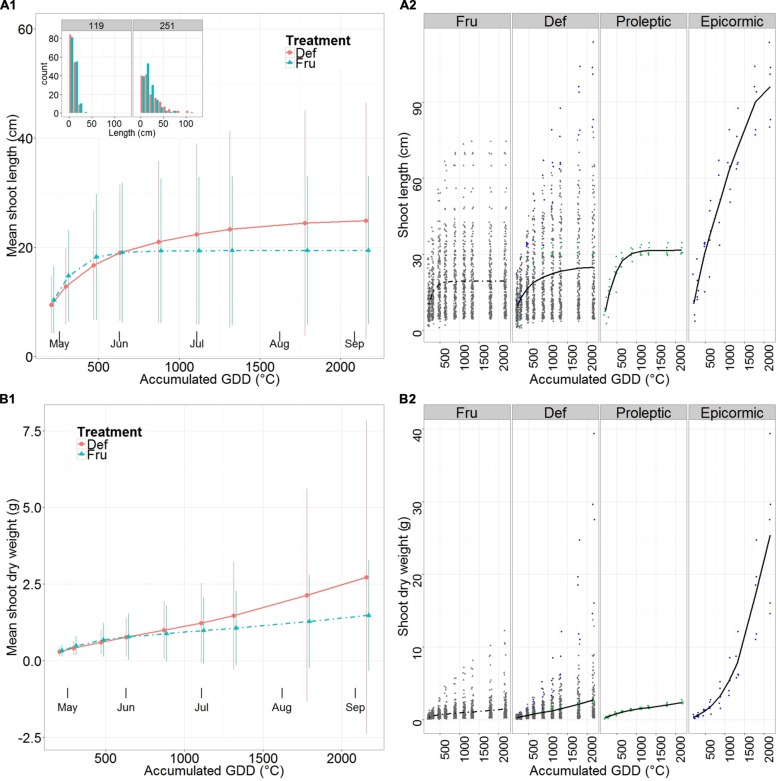
**Mean length **(A)** and predicted shoot dry weight **(B)** of shoots on fruited (FRU) and defruited (DEF) trees vs. the accumulated growing degree days (GDD) after bloom.** A synthetic summary of the mean response for the two treatments (FRU, dotdash; DEF, solid) is given **(A1,B1)**. Vertical lines indicate standard deviations. The frequency distribution of lengths for DEF and FRU shoots, at the beginning and end of the growing season is shown (**A1**, upper left corner). A comparison between FRU, DEF, and the Max proleptic and epicormic shoots extracted from the DEF population is provided with individual observations in the background **(A2,B2)**. In **(A2,B2)** Max proleptic and epicormic shoots are colored respectively in green and blue.

At the beginning of the season (30 days after full bloom), shoot mean length was slightly longer in FRU than DEF, but became 28% longer in DEF than in FRU when elongation was complete (**Figure 2A1**, **Supplementary Table S2**). Mean length of FRU shoots approached a plateau by the beginning of June and completely stopped by early July, while it continued growing in DEF, although with a decreasing vigor, until the end of the season. This resulted in fewer relatively short shoots (between 7 and 30 cm), more long shoots (>30 cm) and the unique development of very long shoots (>100 cm), in DEF compared to FRU.

The Max proleptic and Max epicormic shoots (**Figure 2A2**, **Supplementary Table S2**) grew from similar sizes at the beginning of the season, but while proleptic shoots completed their elongation by early June (31.6 cm ± 1.9 cm), epicormic shoots continued growing until late August (96.2 cm ± 14.3 cm).

**FIGURE 3:**
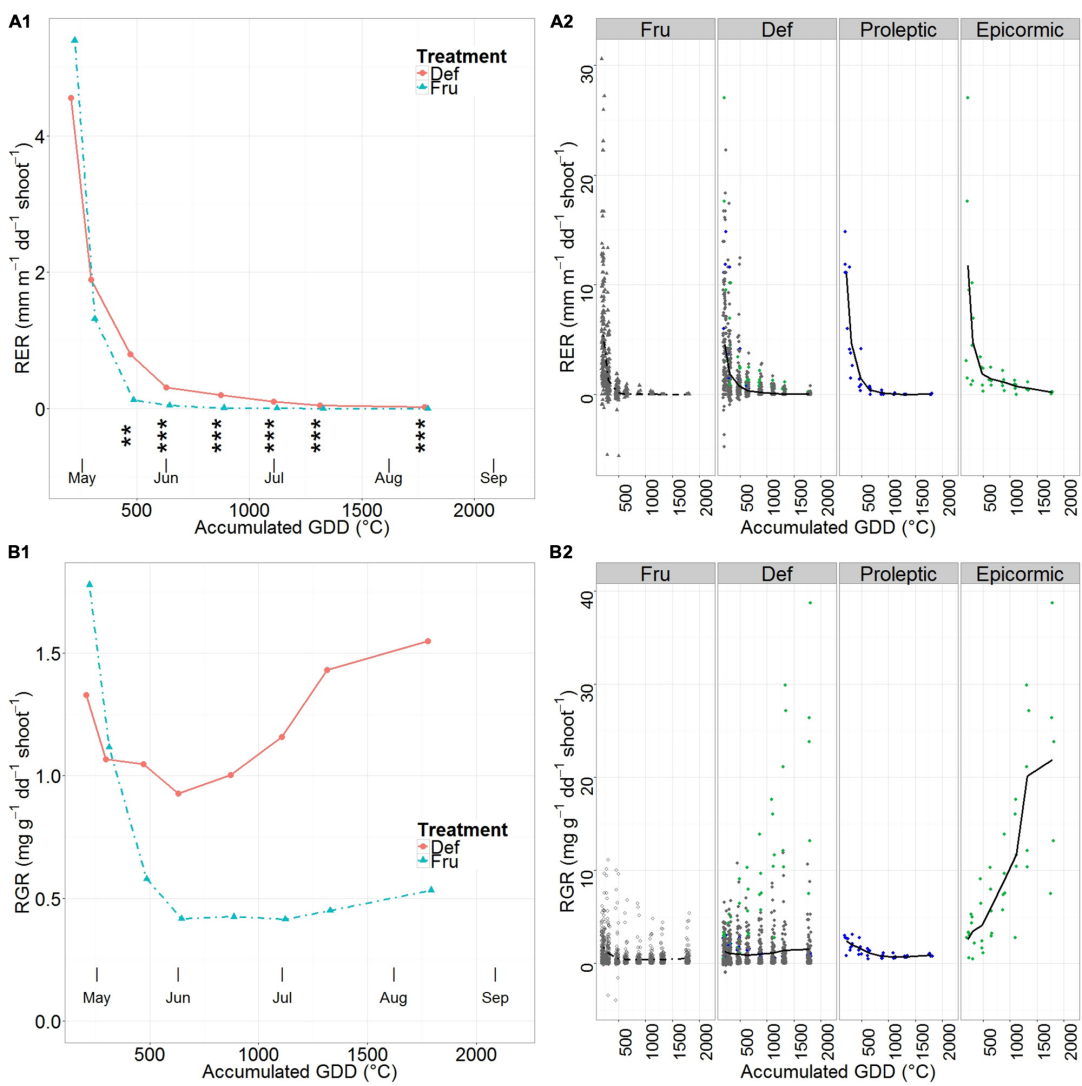
**Mean relative elongation rate (RER) **(A)** and relative growth rate (RGR) **(B)** of shoots on fruited (FRU) and defruited (DEF) trees vs. the accumulated growing degree days (GDD) after bloom.** A synthetic summary of the mean response for the two treatments (FRU, dotdash; DEF, solid) is given **(A1,B1)**. In A1 stars indicate the level of significant differences between RER of DEF and FRU trees (^∗∗^ <= 0.01, ^∗∗∗^ <= 0.001). A comparison between FRU, DEF and the Max proleptic and epicormic shoots, extracted from the DEF population, is provided with individual observations in the background **(A2,B2)**. In **(A2,B2)** Max proleptic and epicormic shoots are colored respectively in green and blue.

Mean shoot RER steeply decreased from the early season until the beginning of June in all groups (**Figure 3A1**). RER started with comparable values for DEF and FRU (**Figure 3A1**, **Supplementary Table S3**), and began to plateau (RER < 10^-2^ mm m^-1∘^C^-1^ stem^-1^) in June for FRU and in mid July for DEF. Differences between DEF and FRU RERs were significant from late May until fruit harvest.

RER of Max proleptic and Max epicormic shoots (**Figure 3A2**, **Supplementary Table S3**) sharply decreased from the beginning of the season until late April, but approached zero in early June for proleptic shoots, and only in late August for epicormic shoots.

### Shoot Growth

Shoot dry weights were estimated both for FRU and DEF trees based on eq. 1. This was done after exploratory analysis on shoot density and secondary growth suggested that an approximation of the biomasses of DEF shoots could be obtained by applying the same eq. 1. First, a multi-variate linear regression model, built between the basal diameters, lengths, treatments and measurement date (mid-July, mid-August, early-September and in the following winter) of 10 tagged shoots for each one of five DEF and FRU trees showed that, on average, DEF shoots always had significantly larger basal diameters, and therefore larger volumes, than FRU shoots of the same length (**Supplementary Table S4**). Second, a one sided *t*-test showed that DEF shoots sampled in the following winter were not significantly more dense than the FRU shoots (**Supplementary Figure S9**). Eventually, the estimation of date specific fruit densities for THI and FRU trees showed that the difference between treatment specific fruit density continuously increased during the season (**Supplementary Figure S10**), suggesting a continuous pattern also in the case of FRU and DEF shoots. This difference was not significant by the end of the season, suggesting no differences through the whole year. As a consequence of these analysis, the biomasses obtained for DEF shoots could be considered lower estimates of their actual values.

The pattern of shoot mean dry weights differed among treatments: growth increased almost linearly for both FRU and DEF, but with an initial inflection in FRU, corresponding to the end of the elongation period. At the beginning of the season mean dry weights differed by less than 10% between treatments, but reached a value 83% higher in DEF than in FRU by the end of the growing season (**Figure 2B1**, **Supplementary Table S5**).

Max proleptic and epicormic shoots had similar dry mass values (**Figure 2B2**, **Supplementary Table S5**) at the beginning of the season, however, while proleptic shoots grew approximately linearly until the end of the season, the epicormic shoots grew exponentially until late June and then linearly until late August.

The common traits in the patterns of mean shoot RGR of FRU and DEF (**Figure 3B1**, **Supplementary Table S6**) were the relatively high initial values followed by a steep drop until early June, a relatively stable phase in June, and a clear increase from early July. Specifically, the mean shoot RGR in FRU dropped by 77%, from the highest values in the early season until the end of the elongation period (early June); remained constant until the beginning of July, and eventually restarted growing linearly until harvest. Mean shoot RGR in DEF also decreased from the beginning of the season until early June, but decreased less than FRU (72% of the initial DEF value); it restarted growing with a steep increase by early July and peaked by harvest (123% of the initial value).

Max proleptic shoots had quite contrasting RGR patterns compared to epicormic shoots (**Figure 3B2**, **Supplementary Table S6**). While RGR was similar for proleptic and epicormic shoots at the beginning of the season, it decreased until early June and then remained relatively constant until late August for the proleptic shoots, while it constantly increased, until the end of the season, for the epicormic shoots.

### Trunk Growth

**FIGURE 4:**
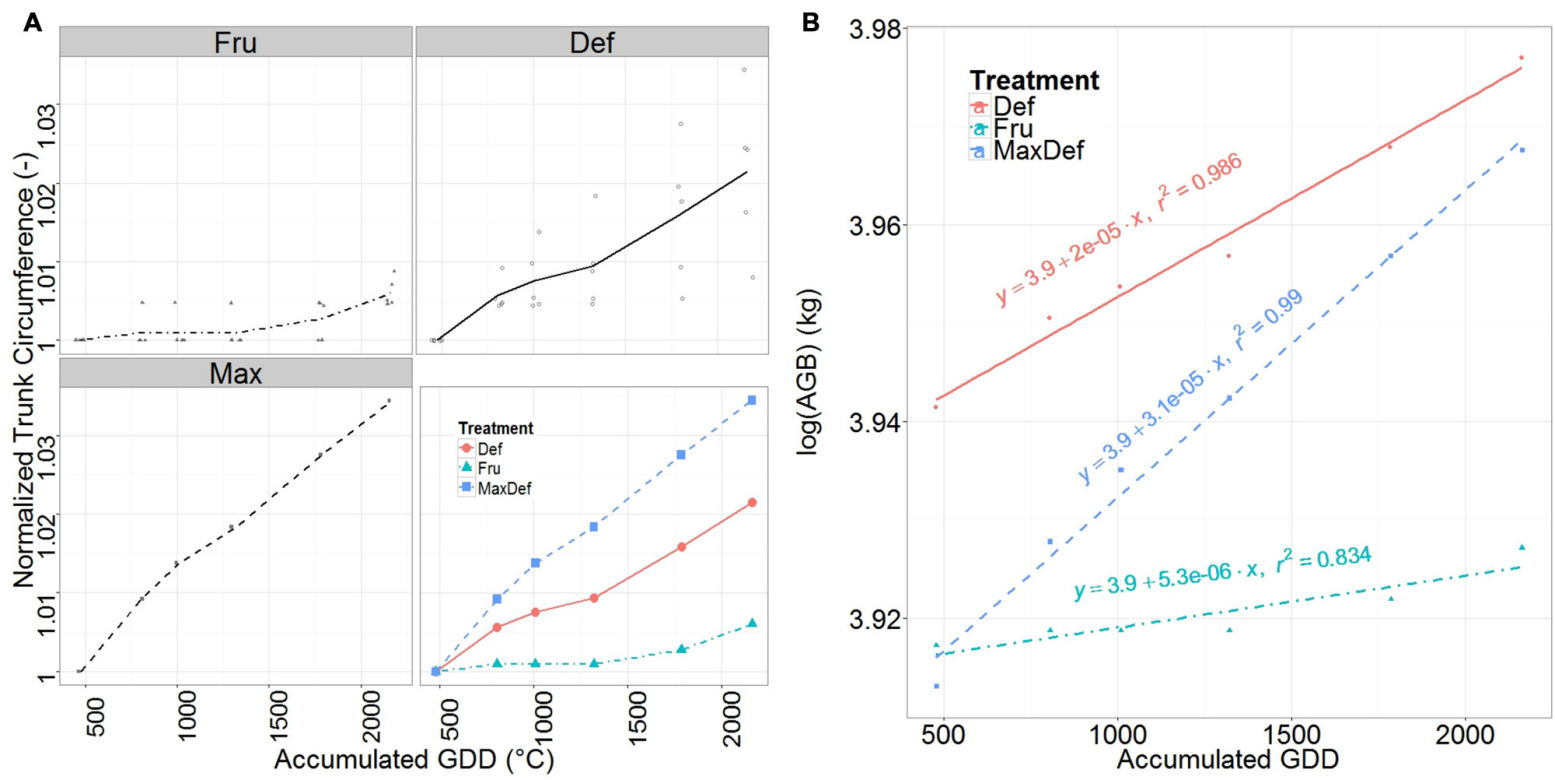
**Mean normalized trunk circumference **(A)** and relative growth rate (RGR) of the above ground dry woody biomass **(B)** for fruited (FRU) and defruited (DEF) trees, and of the Max tree, vs. the accumulated growing degree days (GDD) after bloom.** Individual observations of trunk circumference are shown in the first three panels **(A)**. The fourth panel of **(A)** shows a comparison among the FRU, DEF and Max. Straight lines fitted to the natural logarithm of the total above ground woody biomass are presented in **(B)**. Their slopes indicate their RGRs.

Trunk circumference increased almost linearly from full bloom until the end of the growing season (**Figure 4A**), with similar values for FRU and DEF were found at the beginning of the season, but lower values for FRU than for DEF by the end of the season.

Trunk RGR (corresponding to the slope of the fitted regression line through the values of trunk growth vs. GDD, **Supplementary Table S7**) was 55% higher for the tree with the highest trunk RGR (MaxDEF) than for DEF, and 270% higher for DEF than for FRU (**Figure 4B**). In all cases, a slight decrease in RGR was observed around the end of July.

### Fruit Growth

**FIGURE 5:**
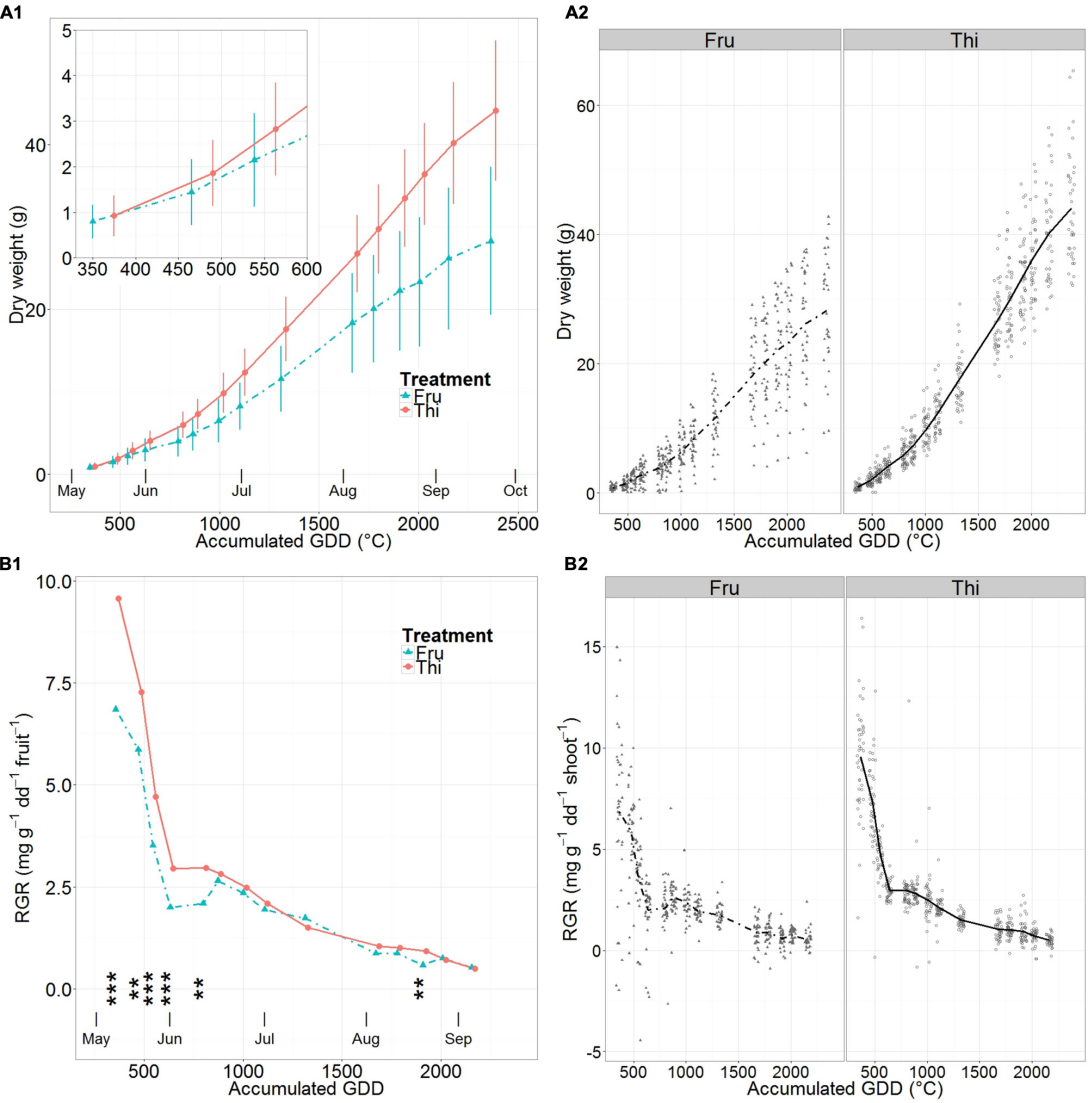
**Mean apple dry weight **(A)** and relative growth rates (RGR) **(B)** on fruited (FRU) and thinned (THI) trees vs. the accumulated growing degree days (GDD) after bloom.** A synthetic summary of the mean response for the two treatments (FRU, solid; THI, dotted) is given **(A1,B1)**. Vertical lines indicate standard deviations. In **(B1)** stars indicate the level of significant differences between RGR of FRU and THI trees (^∗∗^ <= 0.01, ^∗∗∗^ <= 0.001). Fruits that presented pest damage were removed from the dataset. A comparison between FRU and THI, with individual observations in the background, is shown **(A2,B2)**.

Mean fruit dry weight accumulation followed a sigmoidal pattern in all cases (**Figure 5A**, **Supplementary Table S8**). It increased rapidly until about mid-June, then become linear until early September and finally decreased near harvest. The distribution around the mean dry weights were larger in FRU than in THI (**Figures 5A1,2**). In particular, at the beginning of the season (30 days after full bloom) fruit mean dry weights were similar for FRU and THI but differed by 55% at harvest.

Patterns of fruit mean RGR (**Figure 5B1**, **Supplementary Table S8**) were of a rapid linear decrease, from relatively high early season values until early June, followed by a less steep, almost linear decrease until harvest. The RGR values in FRU were markedly lower than THI until mid-June, when they experienced a short but clear increase until late June. At the beginning of the season fruit mean RGRs of THI was 39% higher than in FRU. By the end of the initial phase of sharp drop (early June) THI was still 47% higher than FRU. Then, starting after the short increase in FRU RGR, that occurred by mid-June, the differences among treatments remained always smaller than 20%, except 3 weeks before harvest, when they briefly peaked to 60%. RGR of THI fruit was significantly higher than FRU from the first measurement date until mid-June, and three weeks before harvest.

## Discussion

### Absolute Organs Growth and Periods of Resource Limitation

Stem primary growth in FRU trees stopped by the end of June as in Lauri et al. (2010), while DEF trees had a portion of stems that continued growing, and showed significant summer flushes (**Figure 2A2**). Consistently with previous works (Dudney, 1974; Hansen, 1977; Monselise and Goldschmidt, 1982; Lauri et al., 2010; Martínez-Alcántara et al., 2015) in our experiment stem elongation and growth (**Figures 2A1,B1**, **Supplementary Tables S2** and **S5**) on normally cropped trees were more limited than on trees carrying a lower fruit load (in this experiment the DEF trees).

Regarding the fruit, their mean growth pattern differed slightly from the one described by the ‘expolinear’ model (Lakso et al., 1999) with an initial phase of exponential increase in fruit dry weight, corresponding to the period of cell division, followed by linear growth until maturity (Lakso et al., 1999; Palmer etal., 2003). Indeed, we observed a late season decrease in growth rates similar to those reported by Stanley et al. (2000) and Lauri et al. (2010) for both the FRU and THI fruit loads, even after removal of the fruits presenting pest damage from the dataset (**Figure 5A**). Larger mean individual fruit sizes were attained on trees with low fruit load (THI) compared to trees with larger fruit loads (FRU) as in Lauri et al. (2010).

Relatively little information is available about periods of resource limitation for vegetative growth of apple trees. However, the reported increase in shoot number, length, secondary growth and dry mass, and of summer flushes in trees with decreased fruit loads (Lauri et al., 2010) implies periods of resource limitation sometime during stem elongation and during the second half of summer.

Concerning the duration of the resource limited period for stems elongation, this lasted much longer in our experiment (from 500 GDD until harvest) than in a late maturing peach cultivar (Cal Red) (from 400 to 600 GDD) (Grossman and DeJong, 1995b) (**Figure 3A1**).

Regarding the perdiod of resource limitation for vegetative growth, as our analysis suggested that the increment in dry mass of DEF shoots was generally higher than in FRU shoots (see Results – Shoot Growth), we conclude that the differences in RGR between DEF and FRU stems occurred at least as long as the corresponding RERs (as in Grossman and DeJong, 1995b).

Patterns of stem RER and RGR in FRU were strictly linked (**Figures 3A1,B1**): decreasing together and approximating a minimum at about 500 GDD. On the contrary, the decrease in RER for DEF stems was not immediately reflected in terms of RGR, indicating that the decrease in stem RGR, related to decreased primary growth, was more than compensated by the extension of summer flushes and secondary growth (**Figure 3B1**).

The secondary growth of the main trunk was also found to be resource limited in FRU trees compared to DEF through the whole growing season (**Figure 4B**) (as in Lauri et al., 2010).

Regarding the fruit, in this study significant differences between RGR of FRU and THI were found from the early season until mid-June (**Figure 5B1**), confirming the period of resource limited growth for apples in standard field conditions presented in Hansen (1977).

The closing of RGR gap between FRU and THI in early June suggests the end of a period of resource limitation for fruits in normal field conditions. Apple fruits compete with the vegetative growth for resources, and especially with the nearby shoots (Lauri et al., 2010). In the current experiment, early June was also the time when proleptic shoots stopped elongating (**Figure 2A2**). Concurrently, an overall increase in the vegetative RGR (**Figure 3B1**), largely related to the growth of epicormic shoots (**Figure 3B2**), was observed. This suggests that the end of the elongation of proleptic shoots leaves enough resources available to allow for a significant increase in fruit growth rate (for FRU), shifting the growth of FRU fruits from resource limited to sink limited. A second period of resource limitation for fruit growth was recorded just on one sampling, and occurred approximately 1 month prior to harvest; this might be related to a temporary decrease in photosynthetic active radiation (PAR), that might reduce carbon availability for fruit growth before harvest (Lakso and Corelli Grappadelli, 1998; Lakso et al., 1999).

### Identifying Maximum Potential RGR

The variability in RER/RGR observed in both the THI and DEF treatments suggests that resource availability could have locally limited the growth of some organs, even in trees where competition for resources was reduced by manipulations.

Making the distinction between potential proleptic and epicormic shoots, and extracting the shoots that reached the highest dry mass by the end of the season, allowed discrimination between maximum potential and supposedly sub-optimal shoot growth, and estimation of shoot-type-specific RER and RGR patterns. These were markedly different among shoot types, and likely provided a better approximation of their maximum potential than the mean DEF (**Figures 3A2,B2**). Because of their uninterrupted seasonal increase in RGR, the Max epicormic shoots qualitatively differed from all other organ types. In particular, by becoming large, the stem to leaf dry mass ratio (axialization) of each individual shoot increased (Lauri and Kelner, 2001), suggesting that a relatively large fraction of the assimilated carbohydrates (**Figure 2B2**) was used for the growth of the shoots themselves (Johnson and Lakso, 1985).

While a large part of the fruit variability could simply have been due to different starting times of growth, for stems this was more likely related to mechanisms such as apical dominance and suppression of growth. On the one hand, the concept of semi-autonomy of organs suggests that an environmental factor, such as light availability and consequent locally produced photoassimilates, could explain differences in growth among shoots. On the other hand, endogenous factors such as apical dominance, related to the interactions within the tree structure, may play a major role in the definition of the relative carbon demand of individual shoots. In this context, sugars, and in particular sucrose related molecules, have been recently indicated as important modulators of bud growth, suppression of bud outgrowth and regulators of apical dominance (Barbier et al., 2015a,b). Sucrose has been also found to indirectly adjust phloem unloading, sink strength, and carbon allocation in sink tissues (Roitsch and González, 2004; Barbier et al., 2015a). This suggests that a positive feedback might occur in the determination of the seasonal carbon demand of individual stems, as already proposed for fruit (Minchin et al., 1997). Considering the sugar related compounds as both growth signals and trophic sources, shoot semi-autonomy, and light driven apical dominance may not be alternative, but may be fundamentally interacting factors in the emergence of the tree structure.

Regarding fruit growth, much of the variability in RGR for THI (**Figure 5B2**) was explained by the early fruit dry weight (**Supplementary Figure S8**). This suggests that heavy thinning was quite effective in creating conditions close to sink limited for fruits, resulting in good approximation of their maximum potential RGR (Grossman and DeJong, 1995a).

In order to grow to its maximum potential, an organ is expected to grow at its potential RGR through the whole season (Grossman and DeJong, 1995c). In our experiment, the THI fruits that reached the largest dry weights at harvest had relatively low RGR values and high dry mass (**Supplementary Figure S11**) at the beginning of the season, when compared to the mean THI fruits. This was probably due to their relatively early fruit set, which conferred a longer time of growth. Additionally, by setting earlier, these fruits could experience cooler early season temperatures, which are positively related to longer periods of cell division (Warrington et al., 1999), higher early season dry weights, and thus higher fruit dry weight at harvest (**Supplementary Figure S7**) (Stanley et al., 2000).

### GDD as Predictor for Growth

Although for an ectothermal organism GDD may better represent time compared to chronological time, a linear use of GDD might not realistically represent growth. GDD have indeed been reported to be non-linearly related to environmental temperature. Strong, non-fully reversible, temperature-dependent changes in carbon allocated to young fruit were found for temperatures increasing over 30°C (Minchin et al., 1997). This suggests the existence of a feedback mechanism between temperature, the synthesis of the sucrose synthase enzyme and carbon demand. Also, as related to some developmental processes, e. g., cell division, fruit growth responses are reported to be non-linearly related to temperature at a given time of the year, and to change during the season (Warrington et al., 1999; Stanley et al., 2000; Lopez and DeJong, 2007). These observations suggest that the carbon demand curve of a single fruit might be dependent on both its historical microclimatic conditions and size. Therefore, in order to flexibly represent the seasonal patterns of individual organ carbon demand, maximum potential RGR patterns should be built on the basis of temperature and time dependent non-linear relationships. As such, a clearer, more integrated understanding of these growth dynamics is necessary.

## Conclusion

This study pointed out the importance of analyzing the variability in the growth patterns of different organ types when studying potential growth rates in fruit tree species. First, the periods of resource limitation for vegetative and reproductive organs were identified at the whole plant scale by comparing the RGR of individual organs in trees growing in standard field conditions with DEF and heavily THI tress (Grossman and DeJong, 1995a,c). The resulting mean RGR pattern for fruits on heavily THI trees seemed to well approximate their maximum potential RGR. On the contrary, the variability in RGR for vegetative organs suggested that the compartmental mean could be a relatively inaccurate estimate of its maximum potential. Thus, RGRs of vegetative organs were determined by discriminating between shoot types (proleptic and epicormic shoots) (DeJong et al., 2012) and by accounting for possible localized resource limitation. These results were qualitatively and quantitatively more accurate representions of the maximum potential RGR patterns, and are expected to be of particular importance for source-sink based tree growth models.

## Author Contributions

FR for experimental design, field sampling, data analysis, and drafting the article, PF for supervision on the statistical analysis, MT, TD, and DG for supervision on experimental design, field sampling, and drafting the paper.

## Conflict of Interest Statement

The authors declare that the research was conducted in the absence of any commercial or financial relationships that could be construed as a potential conflict of interest.

## Abbreviations

DEF: defruited
GDD: growing degree days
FRU: fruited
Max: containing the organs with the highest seasonal values of RGR
RER: relative elongation rate
RGR: relative growth rate
THI: thinned
wood_AGB_: above ground dry biomass excluding current year vegetative shoots
