# Supplementary material for: Maximum Growth Potential and Periods of Resource Limitation in Apple Tree
Source: Front Plant Sci. 2016 Feb 29;7:233. doi: 10.3389/fpls.2016.00233 (PMC4770189; doi:10.3389/fpls.2016.00233)
Supplement: Supplementary file 1 [file Data_Sheet_1.DOCX]

Supplementary Material

**Maximum growth potential and periods of resource limitation in apple tree**

Francesco Reyes^*^, Theodore DeJong, Pietro Franceschi, Massimo Tagliavini and Damiano Gianelle

*^*^****Correspondence****: francesco.reyes@fmach.it, Via E. Mach, 1 38010 S. Michele all'Adige (TN) – Italy*

# Supplementary Figures and Tables

## Supplementary Figures


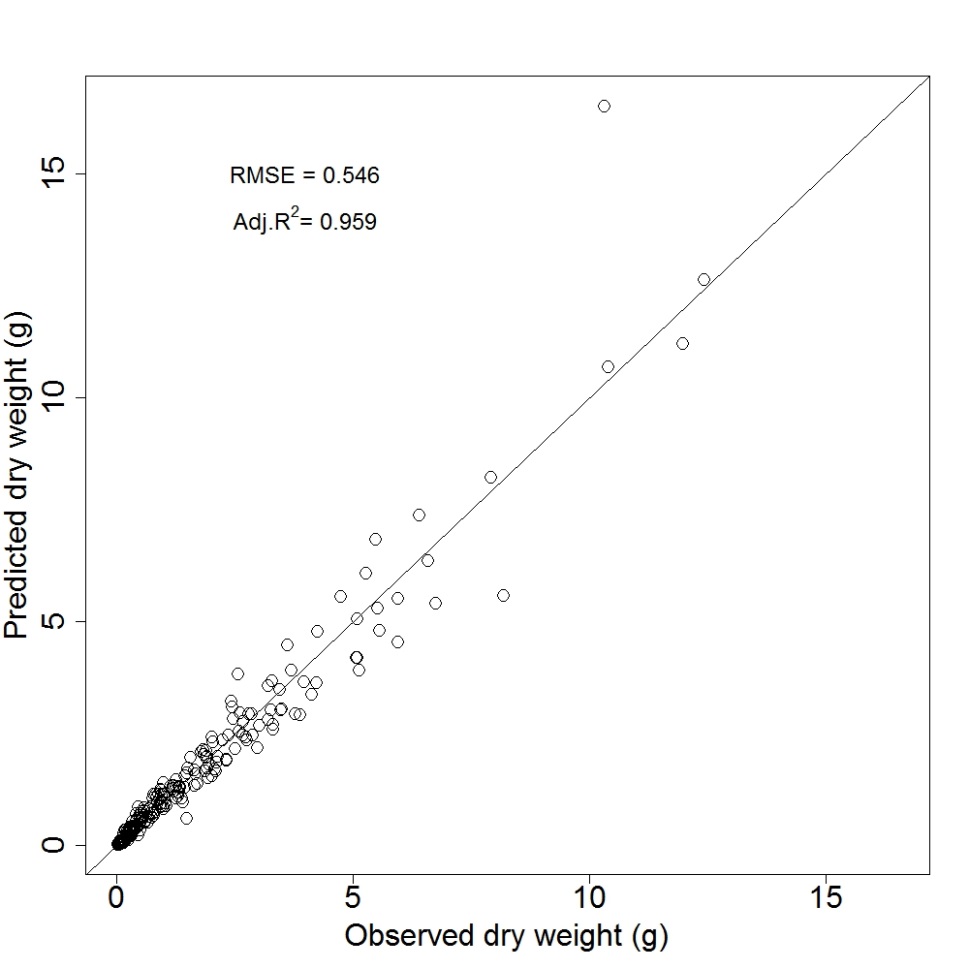


Supplementary Figure 1: Predicted versus observed dry weight of tagged shoots.


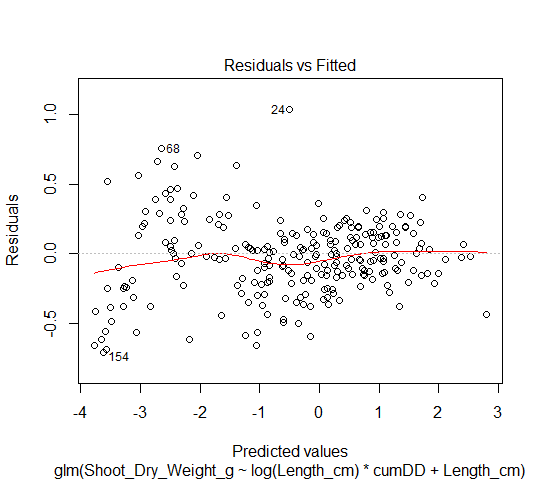


**Supplementary Figure 2:** Distribution of the residual errors between predicted and observed stem dry weights.


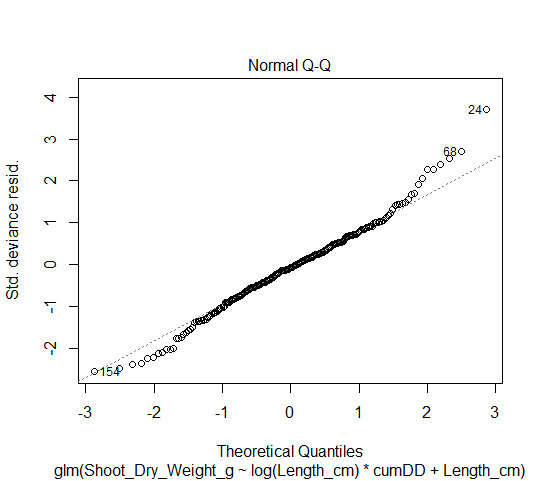


**Supplementary Figure 3:** Q-Q plot of the multivariate model used to predict stem dry weights.


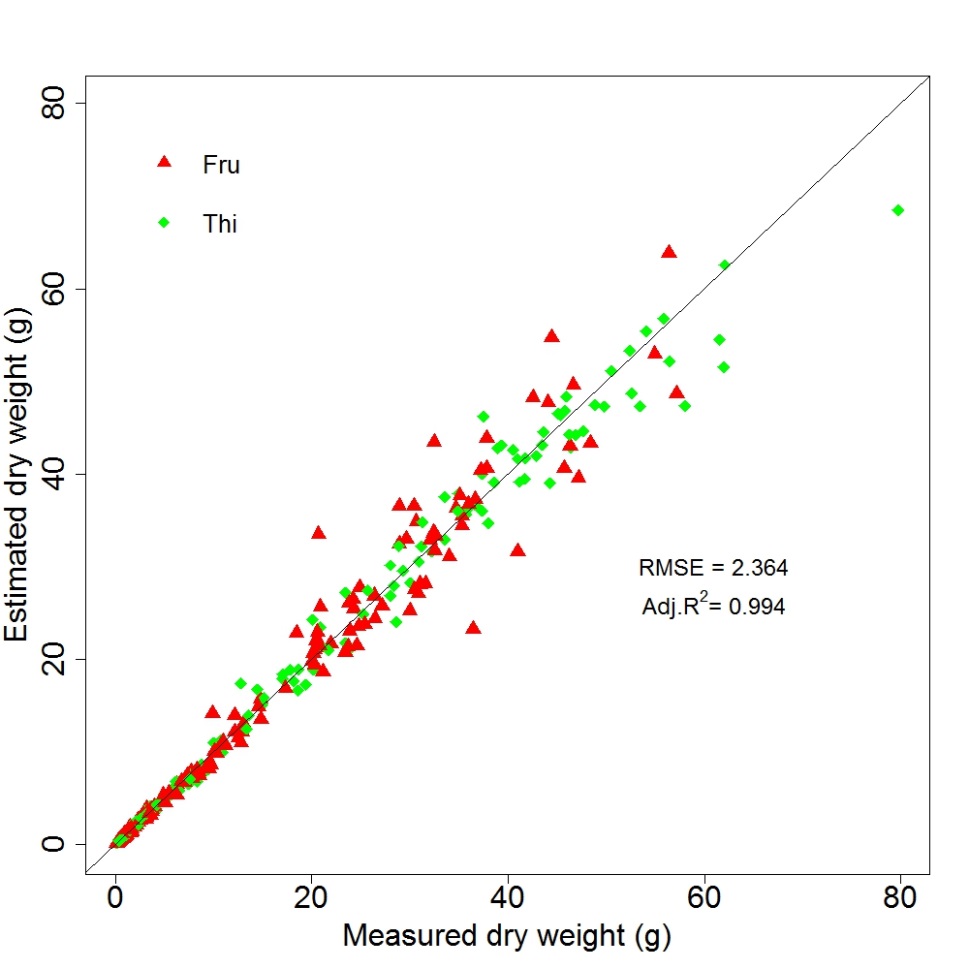


Supplementary Figure 4: Predicted versus observed dry weight of tagged apples on fruited (Fru) and heavily thinned (Thi) trees.


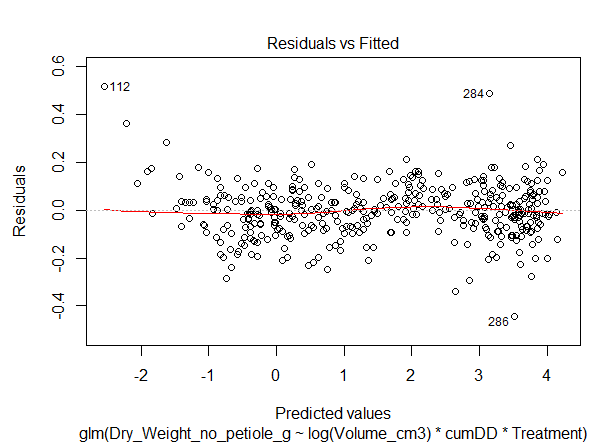


**Supplementary Figure 5:** Distribution of the residual error between predicted and observed apple dry weights.


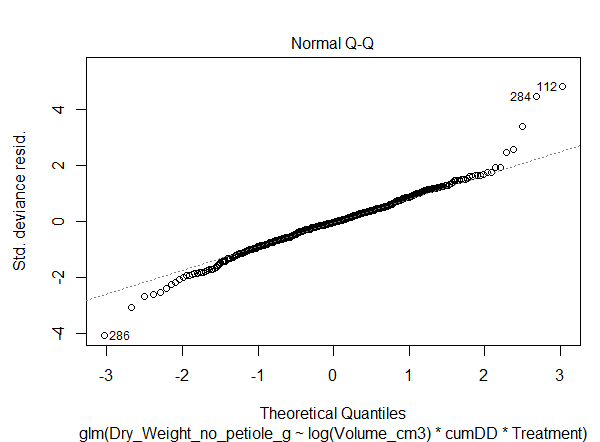


**Supplementary Figure 6:** Q-Q plot of the multivariate model used to predict apple dry weights.


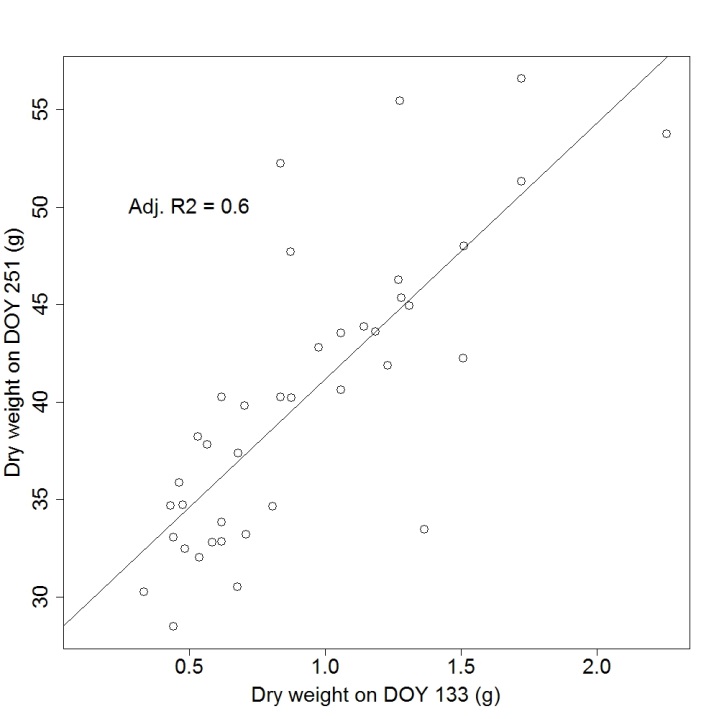


**Supplementary Figure 7:** Fruit dry weight at the beginning vs at the end of the growing season. The straight line indicates the linear model fitted through the two variables.


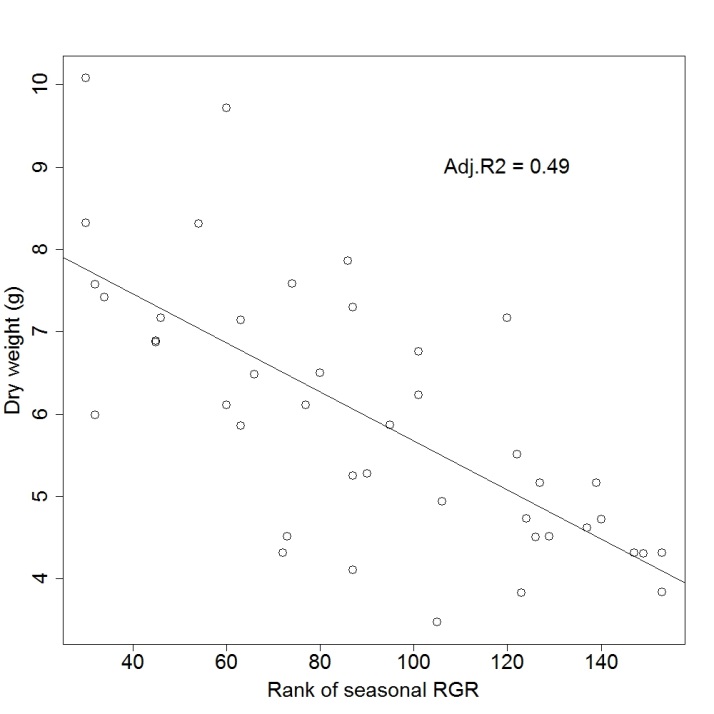


**Supplementary Figure 8:** Rank of the early season individual fruit RGR vs fruit dry weight in early June. High ranks correspond to high early season RGR. The straight line indicates the linear model fitted through the two variables.


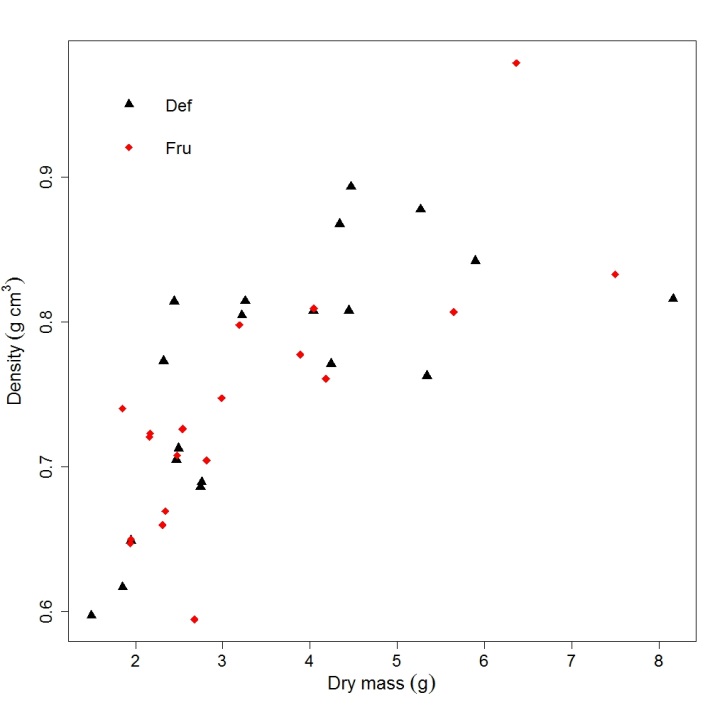


**Supplementary Figure 9:** Dry masses and estimated densities of stems from fruited (Fru) and defruited (Def) trees sampled during the winter following the growing season.


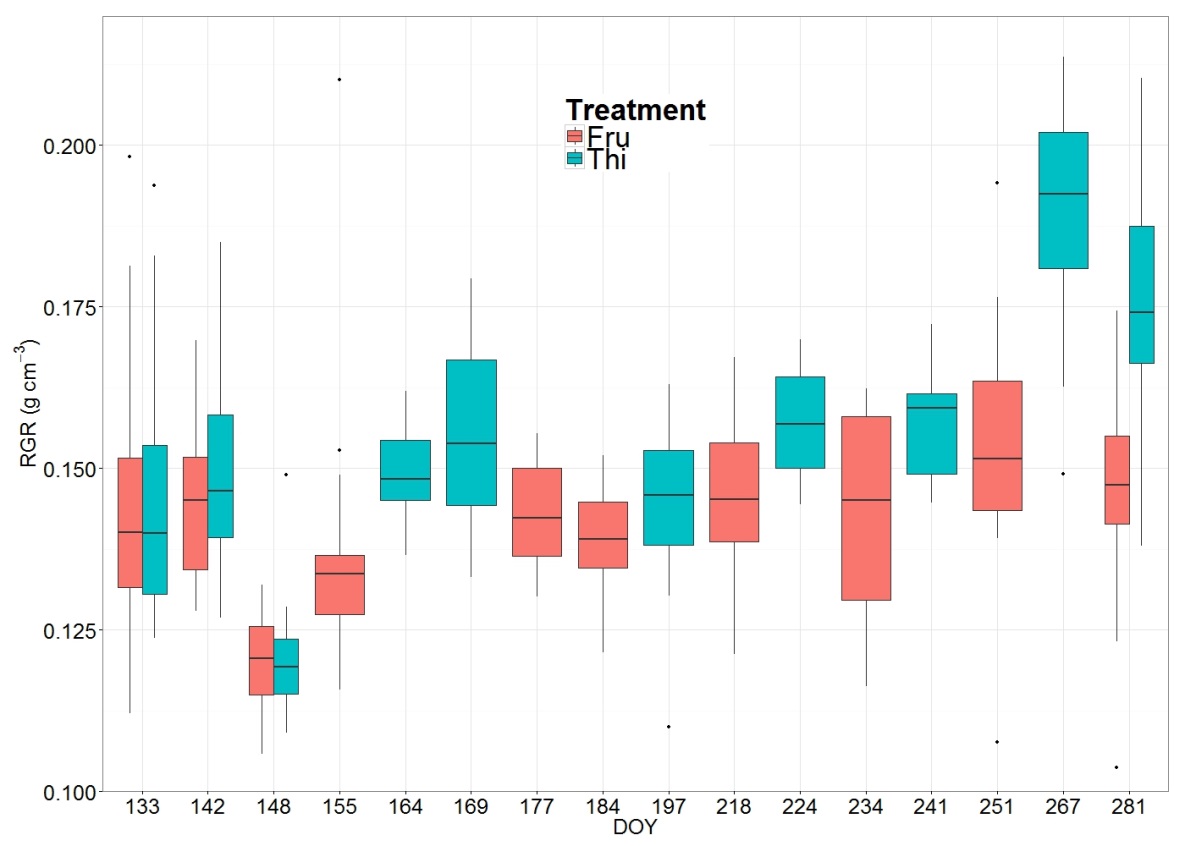


**Supplementary Figure 10:** Estimated density of fruits sampled from fruited (Fru) and heavily thinned (Thi) trees during the growing season (DOY: day of the year).


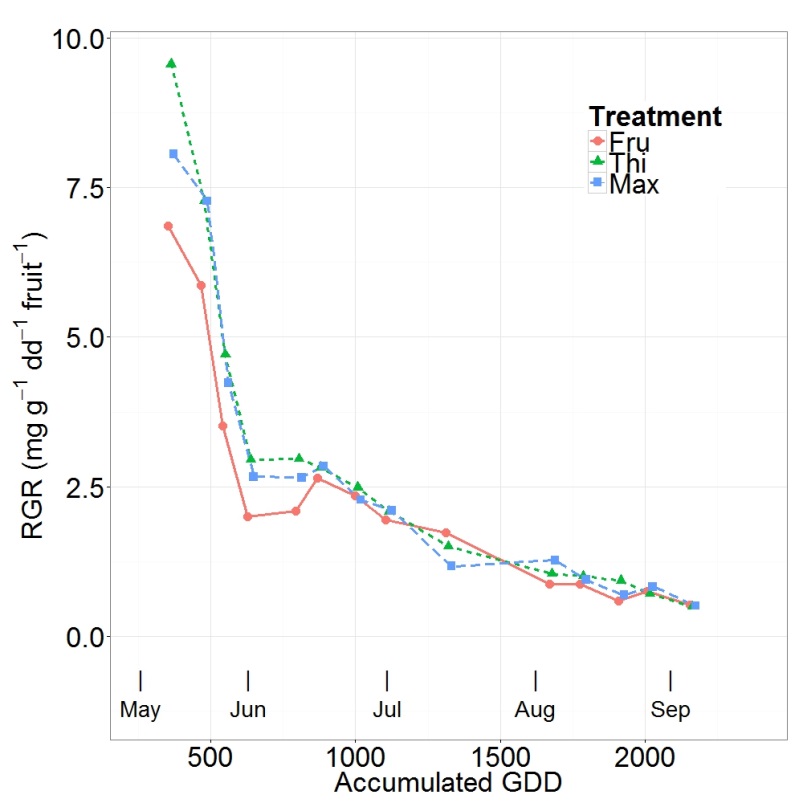


**Supplementary Figure 11:** Mean relative growth rates of apples on fruited (Fru) and thinned (Thi) trees, and the five apples that reached the highest dry mass by harvest (Max), vs the accumulated degree days from bloom.

| Supplementary Tables Supplementary Table 1: Soil nutrient analysis. Organic carbon, total nitrogen, available phosphorus and exchangeble potassium measured at three depths, along and between the rows.   \|  \|  \| Row \| \| \| Inter-row \| \| \| \| --- \| --- \| --- \| --- \| --- \| --- \| --- \| --- \| \| Soil depth \| (cm) \| 0 to 5 \| 5 to 25 \| 25 to 60 \| 0 to 5 \| 5 to 25 \| 25 to 60 \| \| Organic carbon \| C % \| 2.84 \| 1.76 \| 1.19 \| 2.63 \| 1.66 \| 1.2 \| \| Total nitrogen \| N % \| 0.29 \| 0.18 \| 0.11 \| 0.23 \| 0.15 \| 0.11 \| \| Available phosphorus \| P mg/Kg \| 54 \| 52 \| 26 \| 28 \| 36 \| 28 \| \| Exchangeable potassium \| K mg/Kg \| 214 \| 148 \| 110 \| 93 \| 78.6 \| 74 \| |
| --- | --- | --- | --- | --- | --- | --- | --- | --- | --- | --- | --- | --- | --- | --- | --- | --- | --- | --- | --- | --- | --- | --- | --- | --- | --- | --- | --- | --- | --- | --- | --- | --- | --- | --- | --- | --- | --- | --- | --- | --- | --- | --- | --- | --- | --- | --- | --- | --- |

Supplementary Table 2: Shoot length during the growing season. The mean and standard deviation (SD) for fruited (Fru) and defruited (Def) trees, and for proleptic and epicormic shoots extracted from the Def population are shown in respect to the Day of the Year (DOY).

|  | Fru | | Def | | Proleptic | | Epicormic | |
| --- | --- | --- | --- | --- | --- | --- | --- | --- |
| DOY | Length (cm) | SD | Length (cm) | SD | Length (cm) | SD | Length (cm) | SD |
| 119 | 10.3 | 6.2 | 9.5 | 5.2 | 7.5 | 3.8 | 10.7 | 7.1 |
| 128 | 14.8 | 8.4 | 12.9 | 6.9 | 14.3 | 5.7 | 17.6 | 6.5 |
| 142 | 18.2 | 11.5 | 16.7 | 9.9 | 22.9 | 5.1 | 30.1 | 8.5 |
| 155 | 19.1 | 12.8 | 19.0 | 12.5 | 27.4 | 2.4 | 38.7 | 11.7 |
| 169 | 19.3 | 13.2 | 21.0 | 14.8 | 30.3 | 2.2 | 51.1 | 13.6 |
| 184 | 19.4 | 13.5 | 22.4 | 16.6 | 31.1 | 1.8 | 63.1 | 11.0 |
| 197 | 19.4 | 13.6 | 23.3 | 18.0 | 31.4 | 2.1 | 71.8 | 10.1 |
| 224 | 19.4 | 13.6 | 24.5 | 20.7 | 31.4 | 2.1 | 90.0 | 11.6 |
| 251 | 19.4 | 13.6 | 24.9 | 21.7 | 31.6 | 1.9 | 96.2 | 14.3 |

Supplementary Table 3: Shoot Relative Elongation Rate (RER) during the growing season. The mean and standard deviation (SD) for fruited (Fru) and defruited (Def) trees, and for proleptic and epicormic shoots extracted from the Def population are shown in respect to the Day of the Year (DOY).

|  | Fru | | Def | | Proleptic | | Epicormic | |
| --- | --- | --- | --- | --- | --- | --- | --- | --- |
| DOY | RER (mm m^-1^ °C^-1^) | SD | RER (mm m^-1^ °C^-1^) | SD | RER (mm m^-1^ °C^-1^) | SD | RER (mm m^-1^ °C^-1^) | SD |
| 119 | 5.4 | 5.5 | 4.6 | 5.3 | 11.0 | 3.2 | 11.7 | 10.7 |
| 128 | 1.3 | 2.2 | 1.9 | 2.9 | 4.7 | 4.0 | 4.7 | 3.9 |
| 142 | 0.1 | 0.6 | 0.8 | 1.8 | 1.5 | 1.5 | 1.9 | 1.3 |
| 155 | 0.0 | 0.1 | 0.3 | 0.6 | 0.5 | 0.3 | 1.4 | 0.6 |
| 169 | 0.0 | 0.1 | 0.2 | 0.5 | 0.1 | 0.2 | 1.1 | 0.7 |
| 184 | 0.0 | 0.0 | 0.1 | 0.3 | 0.0 | 0.1 | 0.7 | 0.5 |
| 197 | 0.0 | 0.0 | 0.0 | 0.2 | 0.0 | 0.0 | 0.6 | 0.3 |
| 224 | 0.0 | 0.0 | 0.0 | 0.1 | 0.0 | 0.0 | 0.2 | 0.1 |

Supplementary Table 4: Coefficients of the linear model predicting stem basal diameter from stem length, Treatment and day of the year (DOY). Stars indicate level of significancy: *** <= 0.001.

| log(Basal Diameter) = log(Shoot length) + Treatment + DOY | | | | |
| --- | --- | --- | --- | --- |
|  | Coefficient | P value |  |  |
| Intercept | 0.5042698 | 1.39E-14 | *** |  |
| log (Shoot length) | 0.3332974 | < 2e-16 | *** |  |
| Treatment (FRU) | -0.1955248 | < 2e-16 | *** |  |
| DOY | 0.0005363 | 0.000226 | *** |  |

Supplementary Table 5: Shoot dry weight during the growing season. The mean and standard deviation (SD) for fruited (Fru) and defruited (Def) trees, and for proleptic and epicormic shoots extracted from the Def population are shown in respect to the Day of the Year (DOY).

|  | Fru | | Def | | Proleptic | | Epicormic | |
| --- | --- | --- | --- | --- | --- | --- | --- | --- |
| DOY | Dry Weight (g) | SD | Dry Weight (g) | SD | Dry Weight (g) | SD | Dry Weight (g) | SD |
| 119 | 0.3 | 0.2 | 0.3 | 0.2 | 0.2 | 0.1 | 0.3 | 0.2 |
| 128 | 0.5 | 0.3 | 0.4 | 0.2 | 0.5 | 0.2 | 0.6 | 0.2 |
| 142 | 0.7 | 0.5 | 0.6 | 0.4 | 0.8 | 0.2 | 1.2 | 0.4 |
| 155 | 0.8 | 0.7 | 0.8 | 0.6 | 1.1 | 0.1 | 1.8 | 0.8 |
| 169 | 0.9 | 0.9 | 1.0 | 0.9 | 1.3 | 0.1 | 3.3 | 1.5 |
| 184 | 1.0 | 1.1 | 1.2 | 1.3 | 1.5 | 0.1 | 5.4 | 2.0 |
| 197 | 1.1 | 1.2 | 1.5 | 1.8 | 1.7 | 0.2 | 7.8 | 2.7 |
| 224 | 1.3 | 1.5 | 2.1 | 3.5 | 2.0 | 0.2 | 17.1 | 5.7 |
| 251 | 1.5 | 1.8 | 2.7 | 5.1 | 2.4 | 0.2 | 25.4 | 10.3 |

**Supplementary Table 6: Shoot Relative Growth Rate (RGR) during the growing season.** The mean and standard deviation (SD) for fruited (Fru) and defruited (Def) trees, and for proleptic and epicormic shoots extracted from the Def population are shown in respect to the Day of the Year (DOY).

|  | Fru | | Def | | Proleptic | | Epicormic | |
| --- | --- | --- | --- | --- | --- | --- | --- | --- |
| DOY | RGR (mg g^-1^ °C^-1^) | SD | RGR (mg g^-1^ °C^-1^) | SD | RGR (mg g^-1^ °C^-1^) | SD | RGR (mg g^-1^ °C^-1^) | SD |
| 119 | 1.8 | 1.7 | 1.3 | 1.4 | 2.4 | 0.9 | 2.6 | 1.1 |
| 128 | 1.1 | 1.8 | 1.1 | 1.4 | 2.1 | 0.7 | 3.5 | 2.0 |
| 142 | 0.6 | 1.5 | 1.0 | 1.8 | 1.6 | 0.8 | 4.1 | 3.5 |
| 155 | 0.4 | 0.8 | 0.9 | 1.6 | 1.1 | 0.5 | 6.0 | 3.1 |
| 169 | 0.4 | 0.8 | 1.0 | 1.9 | 0.8 | 0.3 | 8.8 | 3.1 |
| 184 | 0.4 | 0.6 | 1.2 | 2.6 | 0.7 | 0.2 | 11.7 | 5.8 |
| 197 | 0.5 | 0.6 | 1.4 | 4.1 | 0.7 | 0.1 | 20.1 | 8.7 |
| 224 | 0.5 | 0.8 | 1.5 | 4.5 | 0.9 | 0.1 | 21.9 | 12.1 |

Supplementary Table 7: Mean dry weight of the above ground woody biomass during the growing season. The mean and standard deviation (SD) for fruited (Fru) and defruited (Def) trees, and for the Max Def tree are shown in respect to the Day of the Year (DOY).

|  | Fru | | Def | | MaxDef | |
| --- | --- | --- | --- | --- | --- | --- |
| DOY | Dry Weight (Kg) | SD | Dry Weight (Kg) | SD | Dry Weight (Kg) | SD |
| 142 | 50.3 | 4.9 | 51.5 | 8.4 | 50.1 | - |
| 164 | 50.3 | 4.9 | 52 | 8.4 | 50.8 | - |
| 177 | 50.3 | 4.9 | 52.1 | 8.5 | 51.2 | - |
| 197 | 50.3 | 4.9 | 52.3 | 8.6 | 51.5 | - |
| 224 | 50.5 | 5.1 | 52.9 | 9 | 52.3 | - |
| 251 | 50.8 | 5.1 | 53.4 | 9.1 | 52.9 | - |

**Supplementary Table 8: Fruit dry weight and Relative Growth Rate (RGR) during the growing season.** The mean and standard deviation (SD) for fruited (Fru) and defruited (Thi) trees are shown in respect to the Day of the Year (DOY).

|  | Fru | | | | Thi | | | |
| --- | --- | --- | --- | --- | --- | --- | --- | --- |
| DOY | Dry Weight (g) | SD | RGR (mg g^-1^ °C^-1^) | SD | Dry Weight (g) | SD | RGR (mg g^-1^ °C^-1^) | SD |
| 133 | 0.8 | 0.4 | 6.9 | 4.1 | 0.9 | 0.4 | 9.6 | 2.7 |
| 142 | 1.4 | 0.7 | 5.9 | 2.2 | 1.9 | 0.7 | 7.3 | 2.1 |
| 148 | 2.1 | 1.0 | 3.5 | 2.2 | 2.8 | 1.0 | 4.7 | 0.8 |
| 155 | 2.9 | 1.4 | 2.0 | 1.4 | 4.0 | 1.2 | 3.0 | 0.4 |
| 164 | 4.0 | 1.9 | 2.1 | 1.1 | 6.0 | 1.6 | 3.0 | 1.5 |
| 169 | 4.8 | 2.0 | 2.6 | 1.0 | 7.3 | 1.8 | 2.8 | 0.8 |
| 177 | 6.5 | 2.6 | 2.4 | 0.9 | 9.8 | 2.4 | 2.5 | 1.0 |
| 184 | 8.2 | 2.9 | 1.9 | 0.5 | 12.4 | 2.8 | 2.1 | 0.5 |
| 197 | 11.5 | 4.0 | 1.7 | 0.5 | 17.6 | 3.9 | 1.5 | 0.3 |
| 218 | 18.3 | 6.0 | 0.9 | 0.7 | 26.7 | 4.7 | 1.0 | 0.5 |
| 224 | 20.1 | 6.5 | 0.9 | 0.6 | 29.7 | 5.4 | 1.0 | 0.4 |
| 234 | 22.2 | 7.2 | 0.6 | 0.5 | 33.5 | 5.9 | 0.9 | 0.6 |
| 241 | 23.3 | 7.9 | 0.7 | 0.3 | 36.4 | 6.2 | 0.7 | 0.4 |
| 251 | 26.1 | 8.6 | 0.5 | 0.5 | 40.2 | 7.4 | 0.5 | 0.3 |
| 267 | 28.3 | 9.0 | - | - | 44.1 | 8.5 | - | - |
